# Supplementary material for: Identification of photoperiod-induced specific miRNAs in the adrenal glands of Sunite sheep (Ovis aries)
Source: Front Vet Sci. 2022 Jul 22;9:888207. doi: 10.3389/fvets.2022.888207 (PMC9354845; doi:10.3389/fvets.2022.888207)
Supplement: Supplementary file 1 [file Data_Sheet_1.ZIP › Supplementary file/miRDeep2 file.zip!/miRDeep2 file/Novel_Structure/LP42A3/Novel_489.pdf]

Provisional ID	:	NC_019481.2_23194
Score total	:	1
Score for star read(s)	:	-1.3
Score for read counts	:	-1.3
Score for mfe	:	2.1
Score for randfold	:	1.6
Score for cons. seed	:	
Total read count	:	9
Mature read count	:	9
Loop read count	:	0
Star read count	:	0

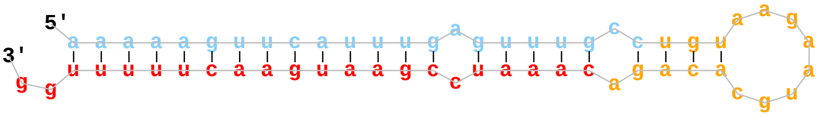

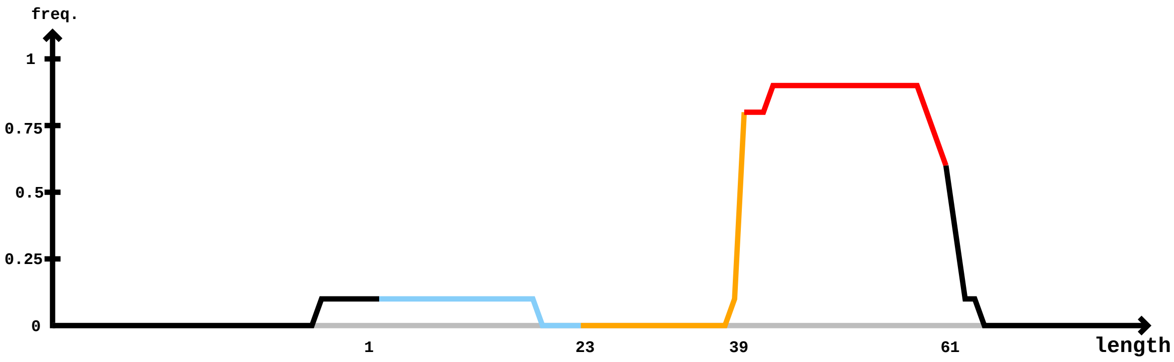

Star   Mature

```
5'- uaagcaaaaagaggcaagacaauggguuggccaaaaaguucauuugaguuugccuguaagaaugcacagacaaauccgaaugaacuuuuuggccaagccaauauuucgcuaa   -3'    exp
    ............(((.(((...(((.((((((((((((((((((((.(((((.((((........)))).))))).)))))))))))))))))))).)))...))).)))..    reads      mm          sample
    ..........................uuggccaaaaaguucauuugagC...............................................................    1          1           seq
    .....................................................................aAaaauccgaaugaacuuuuu......................    1          1           seq
    ......................................................................Aaaauccgaaugaacuuuu.......................    1          1           seq
    ......................................................................Aaaauccgaaugaacuuuuug.....................    1          1           seq
    ......................................................................caaauccgaaugaacuuuuugg....................    1          0           seq
    ......................................................................Aaaauccgaaugaacuuuuugg....................    4          1           seq
    .........................................................................auccgaaugaacuuuuuggUca.................    1          1           seq
```
